# A case of glossopharyngeal neuralgia successfully treated with levetiracetam

**DOI:** 10.1186/s40981-023-00596-x

**Published:** 2023-02-07

**Authors:** Hiroyuki Nishie, Yuka Sakuta, Hideki Nakatsuka

**Affiliations:** Department of Anesthesiology and Intensive Care Medicine, Kawasaki Medical School Hospital, 577, Matsushima, Kurashiki, Okayama, 701-0192 Japan

**Keywords:** Glossopharyngeal neuralgia, Carbamazepine, Levetiracetam

## Abstract

**Background:**

Glossopharyngeal neuralgia is a condition that causes severe pain in the throat during swallowing. Although carbamazepine is a viable option for treating glossopharyngeal neuralgia, there are minimal data regarding the effect of alternative agents to treat it. We report on glossopharyngeal neuralgia, which is successfully controlled by levetiracetam.

**Presentation:**

A woman in her 70s checked into our hospital with a chief complaint of neck pain lasting 5 years. She had a history of carbamazepine-induced interstitial pneumonia. As a result, we prescribed oral levetiracetam 1000 mg daily in addition to mirogabalin, which was previously prescribed. This effectively reduced the numerical rating scale from 9 to 1 with no adverse effects. Finally, she underwent microvascular decompression, and her symptoms were resolved.

**Conclusion:**

Levetiracetam may be an option for patients with glossopharyngeal neuralgia who cannot receive carbamazepine. However, levetiracetam is for off-label use according to the Japanese medical system.

## Background

Glossopharyngeal neuralgia is a condition consisting of sore throat and pharyngeal pain that occurs in the area innervated by the glossopharyngeal nerve owing to vascular compression [1–3]. It is a relatively rare disease, with an incidence of 0.7 per 100,000 population per year [3]. The pain is triggered by coughing, talking, swallowing, or yawning. Like trigeminal neuralgia, it is known to be severe, with the pain lasting from a few seconds to 2 min. Glossopharyngeal neuralgia is often accompanied by vagus nerve irritation, bradycardia, hypotension, and fainting spells. The pain is treated with carbamazepine [1–4] or oxycarbazepine [1, 3], taken orally. Carbamazepine’s side effects include lightheadedness, dizziness, toxic epidermal necrolysis, and interstitial pneumonia. We report a patient who could not take carbamazepine owing to its side effects and was effectively treated with levetiracetam instead. The patient and family have given written consent for the submission of this case for publication. There are no conflicts of interest to report.

## Case presentation

A woman in her 70s checked into our hospital with a chief complaint of left neck pain that had persisted for 5 years. It was induced by drinking water and producing sputum, with a duration of 1–5 min and a refractory period between attacks, and the maximum numerical rating scale (NRS) was 9 out of 10. The pain did not radiate beyond the left neck. Furthermore, she had a history of clipping surgery for a ruptured aneurysm of the left internal carotid artery in her 50s. Although carbamazepine effectively controlled pain, it was withdrawn because interstitial pneumonia occurred 2 weeks after starting treatment. As an alternative, she had taken mirogabalin 10 mg/day orally for 136 days then increased to 15 mg before the initial visit to our hospital.

Blood test results were notable only for a slightly increased white blood cell count of 9880/μL and CRP (C-reactive protein) of 1.10 mg/dL. The previous doctor had not examined the head MRI for pain scrutiny. We started one 500 mg tablet of levetiracetam per day based on a diagnosis of glossopharyngeal neuralgia. According to the Japanese medical system, the use of levetiracetam for neuropathic pain is allowed in our hospital, irrespective of off-label use. Because the pain was reduced 7 days after starting levetiracetam with no side effects, the dose was increased to 1000 mg/day and continued with mirogabalin. Moreover, 50 days later, the NRS was reduced to 0 or 1 out of 10. A brain MRI showed compression of the left glossopharyngeal nerve by the posterior inferior cerebellar artery. The patient was referred to neurosurgeons, and microvascular decompression was performed 59 days after the initial visit to our hospital. The pain completely disappeared after surgery.

## Discussion

Glossopharyngeal neuralgia is usually caused by vascular compression; the posterior inferior cerebellar or the anterior inferior cerebellar arteries often cause compression [1]. It is classified as 13-2 in the International Classification of Headache Disorders [5], and carbamazepine is a representative treatment option [2]. However, carbamazepine can induce severe side effects such as pancytopenia, toxic epidermal necrolysis, and interstitial pneumonia in rare cases [6]. Pregabalin, gabapentin [2], and eslicarbazepin [3] have been reported as alternatives to carbamazepine for glossopharyngeal neuralgia. We did not use pregabalin, which is available in Japan. Pregabalin is a drug similar to the already administered mirogabalin. The difference is that the binding of the α2δ-1 and α2δ-2 subunit of voltage-gated calcium channels (VGCC) by mirogabalin is stronger than that by pregabalin [7], but we deemed that if mirogabalin was ineffective, a drug with a completely different mechanism of action should be preferred. Although the effectiveness of levetiracetam [8], lamotrigine [8, 9], baclofen [8, 9], and phenytoin [2] (suggested as other alternatives for trigeminal neuralgia) for glossopharyngeal neuralgia has not been proved, we utilized levetiracetam, with which we have extensive clinical experience as an anticonvulsant in a palliative care unit.

Levetiracetam has been widely used as an anticonvulsant. Although the detailed mechanism of action is unclear, it binds to synaptic vesicle protein 2A at presynaptic terminals [10], modulating neurotransmitter release and exerting an antiepileptic effect. It is characterized by a very low risk of interaction with multiple drugs [11]. The effectiveness of levetiracetam has been reported for trigeminal neuralgia [8] but not for other neuropathic pain [12]; it might be an alternative in case carbamazepine is contraindicated, as in our case.

In conclusion, we report a case of glossopharyngeal neuralgia that was successfully treated with levetiracetam. Carbamazepine is effective but has many side effects, so it is essential to note levetiracetam as a possible alternative.

## Data Availability

Not applicable.
